# Chronic pain in adults with sickle cell disease is associated with alterations in functional connectivity of the brain

**DOI:** 10.1371/journal.pone.0216994

**Published:** 2019-05-20

**Authors:** Matthew S. Karafin, Guangyu Chen, Nancy J. Wandersee, Amanda M. Brandow, Robert W. Hurley, Pippa Simpson, Doug Ward, Shi-Jiang Li, Joshua J. Field

**Affiliations:** 1 Medical Sciences Institute, BloodCenter of Wisconsin, Milwaukee, Wisconsin, United States of America; 2 Department of Pathology, Medical College of Wisconsin, Milwaukee, Wisconsin, United States of America; 3 Department of Biophysics, Medical College of Wisconsin, Milwaukee, Wisconsin, United States of America; 4 Department of Hematology, Medical College of Wisconsin, Milwaukee, Wisconsin, United States of America; 5 Department of Anesthesia, Wake Forest School of Medicine, Winston Salem, North Carolina, United States of America; 6 Department of Pediatrics, Medical College of Wisconsin, Milwaukee, Wisconsin, United States of America; University of Maryland at College Park, UNITED STATES

## Abstract

Chronic pain affects 50% of adults with sickle cell disease (SCD). Although central sensitization is thought to contribute to the pathogenesis of this chronic pain, no studies have examined differences in functional connectivity of the brain between patients with SCD with and without chronic pain. We performed an observational cohort study using resting-state functional MRI (rsfMRI) of the brain on adults with SCD with and without chronic pain. We tested the hypothesis that, compared to those without chronic pain, those with chronic pain would have differences in functional connectivity between the periaqueductal grey (PAG) and other regions of the brain. Twenty-two adults with SCD, 15 with chronic pain and 7 without chronic pain, as well as 10 African-American controls, underwent rsfMRI of the brain. When SCD patients with chronic pain were compared to those without chronic pain, significant differences in connectivity were noted between the PAG and 9 regions of the brain, including several in the default mode network, a network involved in introspection that has been implicated in other chronic pain syndromes. Changes in functional connectivity between patients with SCD with and without chronic pain suggest a mechanism for chronic pain that involves neuro-plastic changes to the brain.

## Introduction

Chronic pain is prevalent in adults with sickle cell disease (SCD) [1]. Thirty percent of patients experience daily pain, and 50% meet criteria for a chronic pain syndrome [1]. Although it is logical to assume that vaso-occlusion-induced tissue damage (avascular necrosis, leg ulcers) is the cause of this chronic pain [2,3], many patients have widespread pain, or lack an anatomic correlate for their pain, and exhibit hyperalgesia and/or allodynia, signs of peripheral and central sensitization [4–6]. It has been hypothesized that factors inherent to SCD, such as tissue damage, persistent pain input, and inflammation, cause maladaptive changes in the central nervous system (CNS), which promote and sustain the perception of pain and contribute to the pathogenesis of a chronic pain syndrome [7–9]. Recent functional MRI (fMRI) studies of the brain, have demonstrated potentially maladaptive connectivity differences between those with other chronic pain disorders, including fibromyalgia and chronic low back pain, and controls [10–12]. These studies have suggested that the neuro-plastic changes, which links areas of the brain responsible for pain with those responsible for sensation, memory, and emotion, transforms the sensation of pain from a response to noxious stimuli to a neurologic perception in the absence of a stimulus. This “uncoupling” is thought to contribute to the evolution of pain from an intermittent to a chronic phenomenon [13].

Limited data suggest that these neuro-plastic changes occur in patients with SCD [8,9,13,14]. Compared to controls, SCD patients were found to have differences in the default mode network (DMN), insula, and the anterior cingulate cortex (ACC) using electroencephalogram and resting-state (rs)fMRI [9,13]. Another study investigated children with SCD and found associations between changes in the ACC and DMN and rate of acute pain episodes that required hospitalization [8].

In this study, we used rsfMRI of the brain to test the hypothesis that patients with SCD and chronic pain, compared to those without chronic pain, would have differences in connectivity between an area of the brain involved in the suppression of pain, the periaqueductal grey (PAG), and other regions.

## Methods

### Subjects

The institutional review board at the Medical College of Wisconsin and Froedtert Hospital approved this observational cohort study. A convenience sample of African-American adults with SCD was recruited from the Adult Sickle Cell Clinic at Froedtert Hospital. Key eligibility criteria for SCD subjects were: 1) ages 18 to 70 years; 2) any SCD-associated genotype (HbSS, HbSC, HbSbthal) 3) at least 2 exacerbations of pain in the past 12 months requiring contact with medical professionals; 4) no pregnancy; 5) no hematopoietic stem cell transplant; 6) no illicit substances; and 7) no history of stroke or cerebrovascular disease. Key eligibility criteria for controls subjects were: 1) African American; 2) ages 18 to 70 years; 3) no known sickle cell disease or trait; 4) no history of acute or chronic pain disorder; 5) no history of stroke or cerebrovascular disease; and 6) no pregnancy.

For this study, we defined chronic pain as the presence of disease-related pain on ≥ 3 days per week for 6 months [15]. Standard practice in our clinic is to ask the number of days per week that the patient has pain on average to determine the diagnosis of chronic pain. This information was specifically abstracted from medical charts. Patient age, gender, hydroxyurea use, and hospital utilization history was also obtained via the electronic medical record. Lastly, morphine milligram equivalent opioid dose (MME) was calculated from refill records in the electronic medical record.

#### MRI and rsfMRI scanning sessions

Imaging was performed using a whole-body 3T Signa GE scanner with a standard quadrature transmit receive head coil. During the resting-state acquisitions, no specific cognitive tasks were performed, and the study participants were instructed to close their eyes and relax inside the scanner. High-resolution SPGR 3D axial images were acquired for anatomical reference. The number of slices is 144, slice thickness is 1 mm, matrix size is 256 × 192. The sagittal rsfMRI datasets of the whole brain were obtained in 10 minutes with a single-shot gradient echo-planar imaging (EPI) pulse sequence. The fMRI imaging parameters were: echo time (TE) of 25 ms, repetition time (TR) of 2 s, flip angle of 90°; 36 slices were obtained without gap; slice thickness was 4 mm with a matrix size of 64 × 64 and field of view of 24 × 24 cm.

#### rsfMRI preprocessing

Since rsfMRI data is obtained in voxels, the analysis was carried out using the AFNI software and MATLAB programs using SAS 9.4+. The image pre-processing steps included: 1) removal of the first five volumes, 2) motion correction, 3) detrending, 4) removal of averaged signals from white matter and cerebrospinal fluid from each voxel, 5) regression of global signals, and 6) application of a band-pass filter to keep only low-frequency modulations (0.01 Hz to 0.15 Hz). Cardiorespiratory motion artifacts were corrected for using the RETROICOR in the AFNI program. Participant head motion was assessed by evaluating 3 translations and 3 rotations for each scan. Translational thresholds were set to ± 2 mm, whereas rotational thresholds were limited to ± 1°. A subject was excluded from the analysis if head motion exceeded either of these thresholds.

#### Periaqueductal grey (PAG) seed-based functional connectivity (FC) analysis

The PAG, an area of the midbrain that surrounds the cerebral aqueduct, is well known for its role in pain modulation [16]. A descending inhibitor of pain signals, the PAG activates neurons in the brainstem, which in turn activate descending, serotonin-releasing neurons that interfere with ascending signals in the dorsal horn of the spinal cord, where nociceptive signals from the periphery are processed [16]. Beyond its role in blunting pain through its connections in the brainstem, the PAG is also connected to brain regions that are related to executive and emotive functions, such as the prefrontal cortex, the striatum, and the hippocampus [17,18]. It is through these locations that the PAG’s roles in fear, anxiety, and cardiovascular responses are thought to be mediated [17]. Taken together with its role in pain modulation, these executive and emotive functions have led investigators to postulate that the PAG is an integration center for behavioral and autonomic responses to pain. Likely, that is why abnormalities in the PAG have been demonstrated in other chronic pain conditions, including fibromyalgia [11].

Based on these data, we chose the PAG as our “seed.” From there, regions of interest (ROIs) were used to construct the PAG functional connectivity networks. The PAG ROI were separately extracted with the Montreal Imaging Institute (MNI) coordinates (1, -29, -12) provided by a previous study [12]. It was employed as “seeds” for functional connectivity analysis. The ROI seed is 4mm diameter sphere around the coordinates. Then the ROI were co-registered to the functional data (3dfractionize, AFNI). For each subject, the average time course of each seed was extracted from the functional EPI images, and then, was correlated with the time courses of whole-brain voxels, using Pearson cross-correlation (i.e. product-moment). Because the spatial resolutions of the SPGR images (1*1*1 mm) and EPI images (3.75*3.75*4 mm) were different, only those voxels in the EPI images that were occupied at least 50% by ROI voxels masked on the 3D SPGR images were included in the voxel time course analysis [19]. Next, the correlation map was converted to a *z*-value map [Fisher r-to-z transformation, *m* = 0.5*ln(1+r)/(1−r)]. Finally, the data was spatially normalized to the MNI template image, resampled to 2-mm isotropic voxels and smoothed with a 6-mm Gaussian kernel. The individual connectivity map was obtained.

### Statistical analysis

#### rsfMRI data

Individual connectivity maps of the PAG ROI for each subject were analyzed with a one-sample *t-*test to identify voxels. This showed a significant positive or negative correlation with the seed time series, and the pattern of each PAG network for each group was obtained, respectively. To examine the group difference of the PAG network connectivity across all subjects, a voxel-wise, two-sample t-test was performed (*p* < 0.05, corrected with AlphaSim, cluster size > 3060 mm^3^).

First, single sample t-tests were performed using CPM magnitude as a regressor of interest to determine common correlates regardless of clinical status. Second, connectivity was compared separately in the 3 groups. Third, an interaction was performed, in which connectivity was compared across the 3 groups; in this case, the test was for clusters whose connectivity had a differential effect on CPM across groups. Significance was set at P < 0.001 voxel-level, and P < 0.05 cluster-level family-wise error (FWE)-corrected (AFNI 3dClustSim, version 19.0.26, https://afni.nimh.nih.gov/afni, was used for the cluster correction). Significant clusters were extracted and further analyzed in SPSS.

Lastly, in order to account for the possibility that factors unrelated to pain, such as anemia, influence functional connectivity, we performed a regression analysis addressing the relationship in functional connectivity between PAG and hemoglobin by the following model:

$$[PAGfc\;=\;\beta_{0}+\beta_{1}Hgb+\beta_{2}{Group}_{SCD/CN}+\;\varepsilon ].$$

#### Phenotypic data

Median and interquartile ranges (IQR) were used as a summary of continuous or ordinal variables, and frequency variables were summarized as a percent. Groups were compared using a Fisher’s exact test for frequency variables, and an Independent-samples Mann-Whitney U Test was used to determine the significance between groups. A two-sided P-value of <0.05 was considered as significant. Statistical analyses were done by using SPSS 23 for Windows.

## Results

### Subject demographics

We obtained resting rsfMRI data from 32 adults, 22 with SCD (median: 28 years, IQR: 25–37 years) and 10 healthy African-American controls (median: 31 years, IQR: 24–41 years). In comparison to controls, patients with SCD had a lower hemoglobin, and a higher reticulocyte percent (Table 1).

**Table 1:** Demographics for control subjects and patients with SCD with and without chronic pain.

	Sickle Cell Disease	Control Group		Sickle Cell Disease- Pain phenotype		
Demographics	Overall (n = 22)	No chronic pain (n = 10)	P-value	Chronic pain (n = 15)	No chronic pain (n = 7)	P-value
Age, years, median (IQR)	28 (25–37)	31 (24–41)	0.7	31.4 (26–43)	24.1 (21–27)	0.002
Gender, n (% female)	13 (59)	5 (50)	0.7	10 (67)	3 (43)	0.4
SCD genotype (n, %)						
HbSS	17 (77)	NA	NA	10 (67)	7 (100)	0.4
HbSC	4 (18)			4 (27)	0 (0)	
HbSbthal^0^	1 (5)			1 (7)	0 (0)	
Hydroxyurea use, n (% yes)	8 (36)	NA	NA	7 (47)	1 (14)	0.2
Baseline Labs, median (IQR)						
WBC (#/ul)	9.1 (5.6–10.9)	6.3 (4.7–8.3)	0.08	9.1 (5.3–10.5)	8.2 (5.7–12.1)	0.9
Hgb (g/dl)	9.3 (8.5–10.5)	13.8 (11.8–15.8)	<0.0001	9.5 (9.1–10.6)	8.5 (8.2–9.1)	0.01
reticulocyte percent (%)	7.0 (5.5–14.0)	1.5 (1.2–1.8)	<0.0001	7.0 (4.8–12.6)	6.9 (5.9–17.6)	0.5
Hospital Utilization (median number over last 3 years)						
Unscheduled Clinic Visit	1.5 (0–8.3)	NA	NA	1 (0–13)	2 (1–6)	0.8
ED Visit	3 (1–6.3)	NA	NA	3 (1–8)	4 (1–5)	0.8
Inpatient Hospitalization	3 (1.8–6)	NA	NA	3 (1–6)	3 (2–6)	0.8
Morphine milligram equivalent (mg), median (IQR)	72.3 (50.3–240)	NA	NA	190.7 (72.3–317.1)	33.7 (6.7–62.1)	0.002

In the SCD group, 15 (68%) met the criteria for a chronic pain syndrome. When comparing those with SCD and chronic pain to those without, patients with chronic pain were significantly older (P = 0.002), had a higher hemoglobin (P = 0.01), and higher daily opioid use (P = 0.002) (Table 1).

### rsfMRI analysis

**Fig 1:**
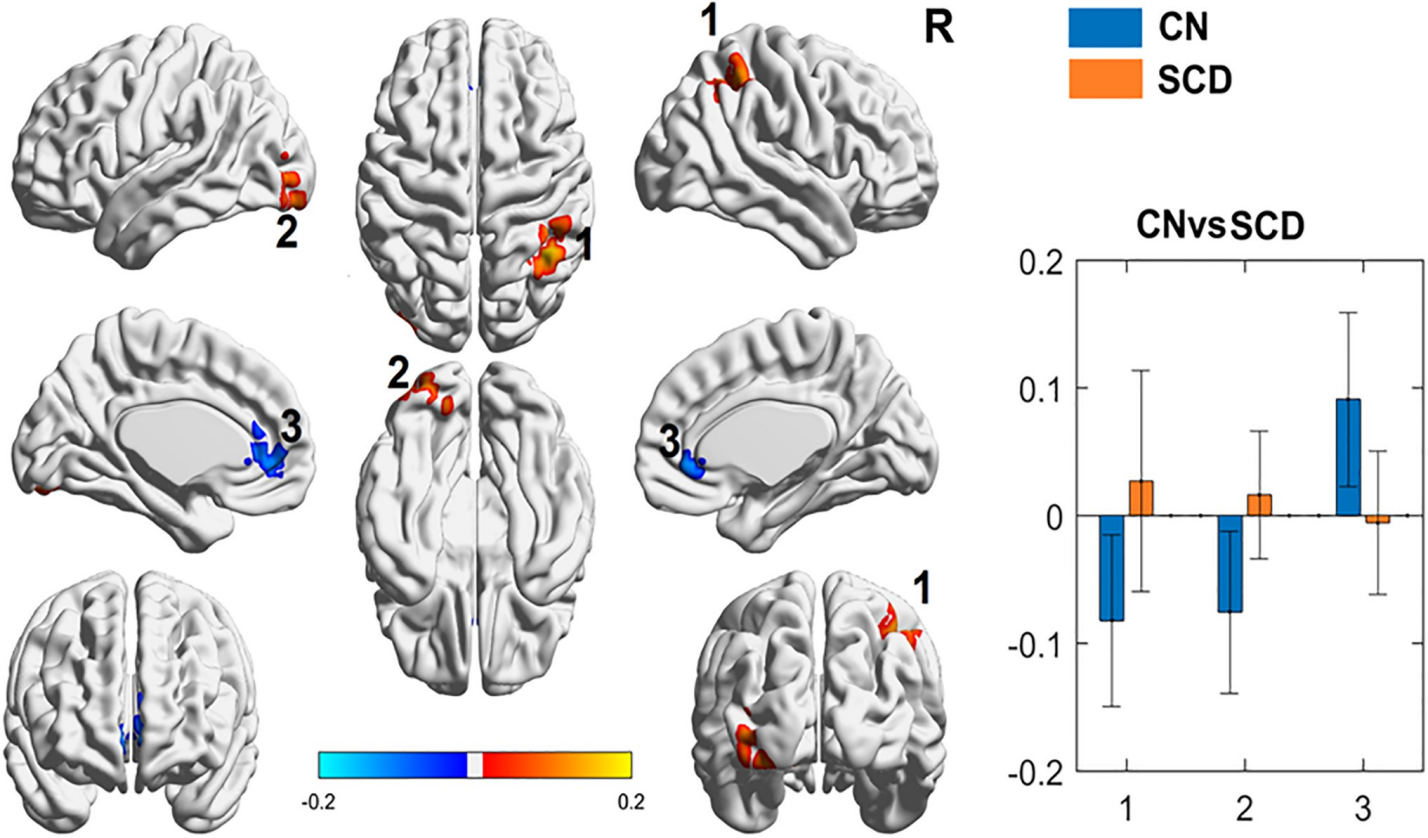
Periaqueductal gray (PAG) seed-based functional connectivity (FC) analyses between patients with SCD (SCD) and controls (CN). *Left*: Images show the results of the two sample t-test pattern of CN (controls, blue) and SCD (patients with sickle cell disease, orange) PAG FC, respectively. Color is coded based on z-score of the significance. Brain regions with warm color represent the positive connection and cold color represents the autocorrelation with PAG regions. Brain regions are numbered: (1) right inferior and superior parietal lobule, (2) left inferior occipital gyrus (Brodmann area 17 and 18), and (3) left and right anterior cingulate. *Right*: In comparison to CN (blue), SCD (orange) demonstrated increased connectivity in (1) right inferior and superior parietal lobule, (2) left inferior occipital gyrus (Brodmann area 17 and 18), and decreased connectivity in the (3) left and right anterior cingulate (y-axis = z-score of the significance, x-axis = brain region of interest by number).

**Fig 2:**
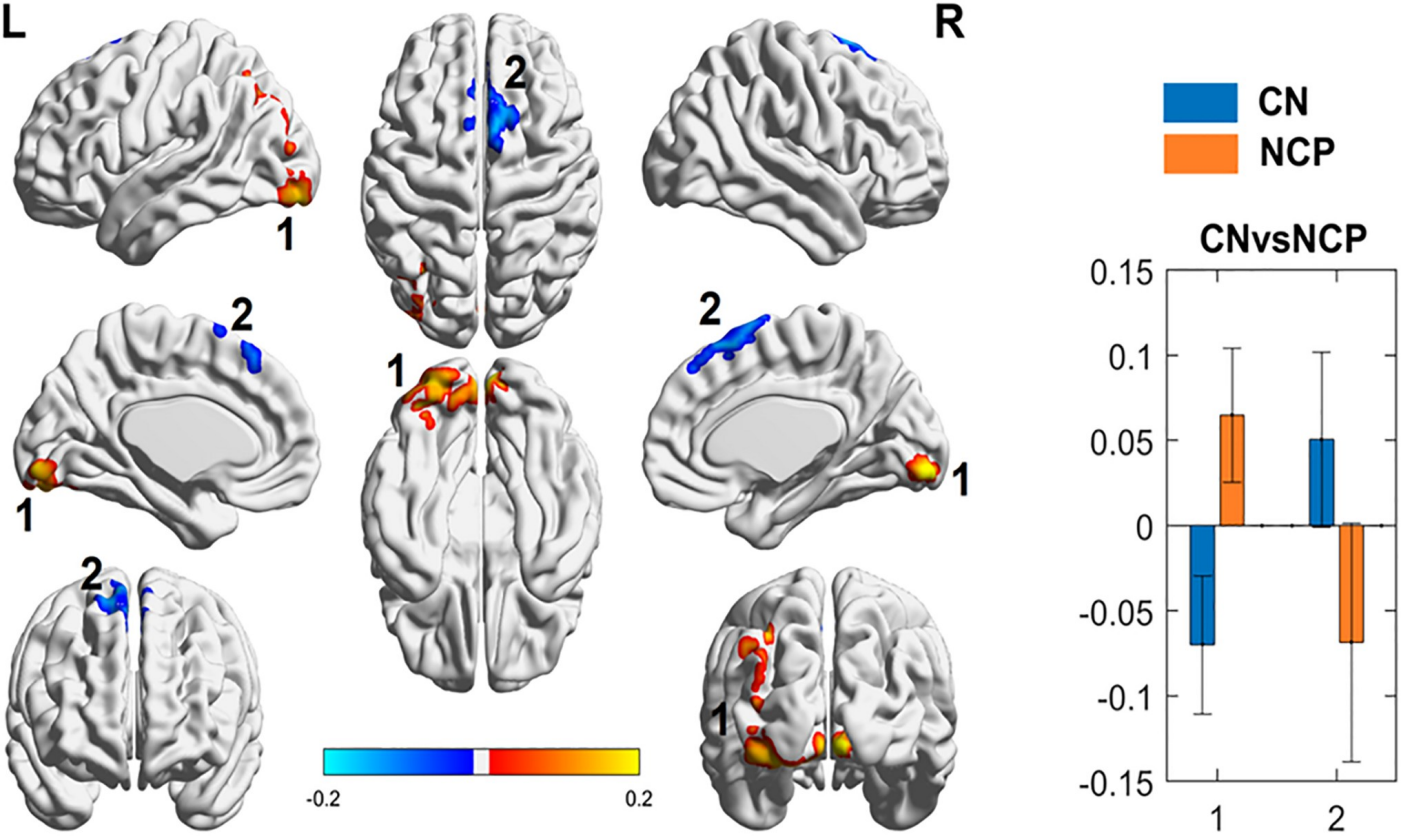
Periaqueductal gray (PAG) seed-based functional connectivity (FC) analyses between patients with SCD without chronic pain (NCP) and controls (CN). *Left*: Images show the results of the two sample t-test pattern of CN (controls, blue) and SCD without chronic pain (patients with sickle cell disease and no chronic pain, orange) PAG FC, respectively. Color is coded based on z-score of the significance. Brain regions with warm color represent the positive connection and cold color represents the autocorrelation with PAG regions. Brain regions are numbered: (1) Left and right Cuneus/ left inferior occipital gyrus (brodmann area 18)/ left middle occipital gyrus/ left and right lingual gyrus, and (2) Left and right superior frontal gyrus. *Right*: In comparison to CN, NCP demonstrated increased connectivity only in the (1) Left and right Cuneus/ left inferior occipital gyrus (brodmann area 18)/ left middle occipital gyrus/ left and right lingual gyrus, and decreased connectivity of the (2) Left and right superior frontal gyrus (y-axis = z-score of the significance, x-axis = brain region of interest by number).

Compared to control subjects, adults with SCD (both with and without chronic pain) had decreased functional connectivity between the PAG and the anterior cingulate cortex (ACC). There was increased functional connectivity between the PAG and the occipital gyrus, and the PAG and the parietal lobe (Fig 1). The change identified in the left middle occipital gyrus could be attributed to the differences in hemoglobin between groups (S1 Fig). When specifically looking at z-scores, healthy subjects show non-zero connectivity while SCD subjects show connectivity that is indistinguishable from zero (Fig 1). When patients with SCD without chronic pain were compared to controls, the only differences in connectivity that were found were between the PAG and the occipital gyrus, and the PAG and the superior frontal gyrus (Fig 2**)**.

**Fig 3:**
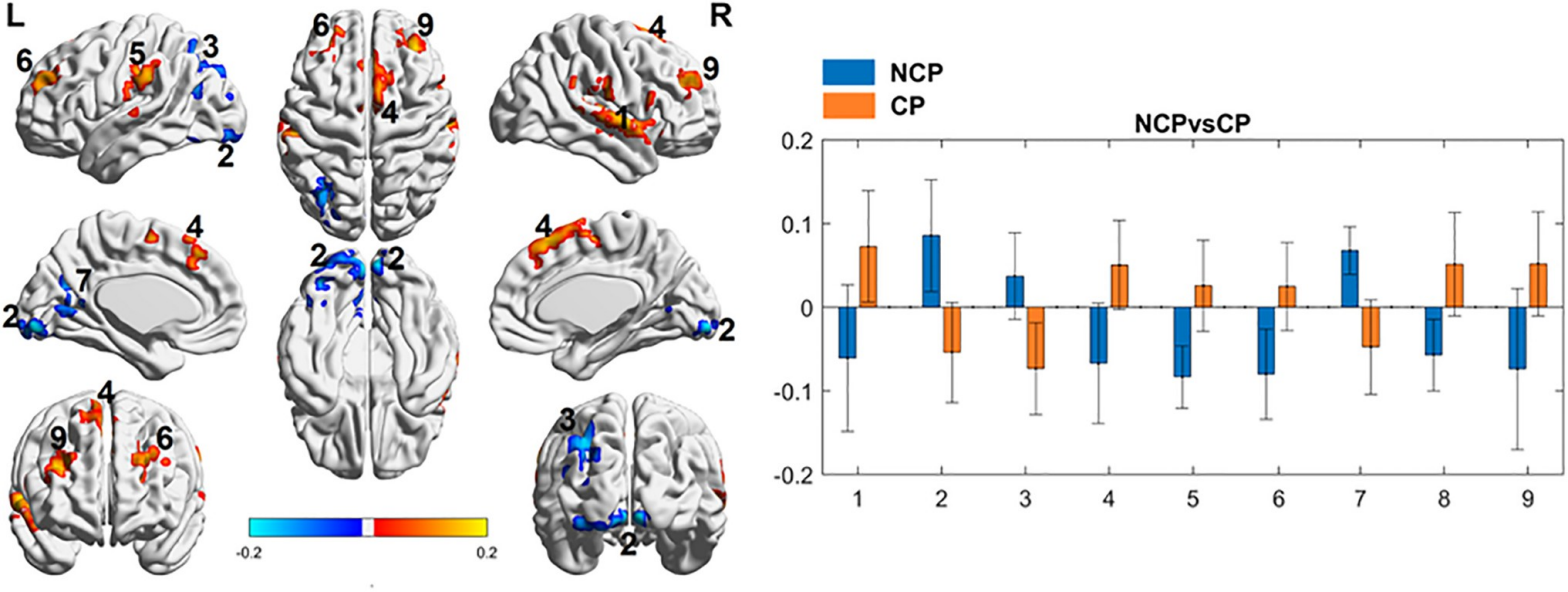
Periaqueductal gray (PAG) seed-based functional connectivity (FC) analyses between patients with SCD with chronic pain (CP) and without chronic pain (NCP). *Left*: Images show the results of the two sample t-test pattern of NCP (SCD patients without chronic pain, blue) and patients with chronic pain (SCD patients with sickle cell disease and chronic pain, orange) PAG FC, respectively. Color is coded based on z-score of the significance. Brain regions with warm color represent the positive connection and cold color represents the autocorrelation with PAG regions. Brain regions are numbered: (1) right superior temporal gyrus/ right insula/ right postcentral gyrus/ right inferior parietal lobule, (2) left lingual gyrus/ left inferior occipital gyrus/ left middle occipital gyrus, (3) left middle temporal gyrus/ left precuneus/ left inferior parietal lobule/ left superior parietal lobule, (4) left and right superior frontal gyrus (right most)/ medial frontal gyrus, (5) left supramarginal gyrus, (6) left superior frontal, (7) left posterior cingulate cortex, (8) right culmen of the cerebellum, and the (9) right middle and superior frontal lobe. *Right*: In comparison to NCP, patients with chronic pain demonstrated increased connectivity in the (1) right superior temporal gyrus/ right insula/ right postcentral gyrus/ right inferior parietal lobule, (4) left and right superior frontal gyrus (right most)/ medial frontal gyrus, (5) left supramarginal gyrus, (6) left superior frontal, (8) right culmen (cerebellum), and the (9) right middle and superior frontal lobe. Decreased connectivity was noted in the (2) left lingual gyrus/ left inferior occipital gyrus/ left middle occipital gyrus, (3) left middle temporal gyrus/ left precuneus/ left inferior parietal lobule/ left superior parietal lobule, and (7) left posterior cingulate cortex (y-axis = z-score of the significance, x-axis = brain region of interest by number).

Next, we compared SCD patients with chronic pain to those without chronic pain. We identified differences in connectivity between PAG and numerous areas, including 13 areas involved with sensory processing, 5 areas involved in motor processing, 7 areas involved in emotion, and 5 areas involved in memory (Table 2, Fig 3). The right/left inferior parietal lobule, left precuneus, medial frontal gyrus, and left posterior cingulate cortex are known components of the DMN (Table 2). For patients with SCD and chronic pain, most areas show functional connectivity that is indistinguishable from zero (except area 1 and 3), and for those same areas, those without chronic pain show a non-zero connectivity (Fig 3).

**Table 2:** Brain regions and their functions where differences in functional connectivity were observed between SCD adults with and without chronic pain.

Function	Sensory processing	Motor processing/ executive function	Emotion	Memory/Learning
Brain Region	Right superior temporal gyrus	Right insula	Right superior temporal gyrus	Right cerebellum
	Right insula	Right/left superior frontal gyrus	Right insula	Left lingual gyrus
	Right postcentral gyrus (i.e. 1° somatosensory cortex)	**Medial frontal gyrus**	**Right/left inferior parietal lobule**	**Left precuneus**
	Right/left inferior parietal lobule*	Right cerebellum	Right cerebellum	Left superior parietal lobule
	Right/left superior frontal gyrus	**Left precuneus**	Left lingual gyrus	**Left posterior cingulate cortex**
	Left lingual gyrus		**Left precuneus**	
	Left inferior occipital gyrus		**Left posterior cingulate cortex**	
	Left middle occipital gyrus			
	Left middle temporal gyrus			
	**Left precuneus**			
	Left inferior parietal lobule			
	Left superior parietal lobule			
	Left supramarginal gyrus			

*Bold = regions associated with the default mode network (DMN).

## Discussion

We demonstrate that a subset of patients with SCD undergo a neuronal re-organization of the brain that is consistent with a mechanism of central sensitization and could explain how chronic pain develops and is sustained in this patient population. Compared to controls, patients with SCD showed few differences in connectivity between the PAG, a region of the brain involved in pain modulation, and other regions; even fewer differences were found when controls were compared to the subset of SCD patients who did not have chronic pain. However, when SCD patients without chronic pain were compared to those with chronic pain, multiple differences in functional connectivity were identified. Several of these brain regions have functions that have been implicated in chronic pain, such as memory, emotion, and introspection, and are part of the DMN, a network whose altered dynamics is thought to be critical for the development of chronic pain syndromes [13,19–25]. These results suggest that, although the pathogenesis of vaso-occlusion may be unique to SCD, the mechanism of chronic pain is not.

In comparison to other chronic pain disorders, such as fibromyalgia [10,11,25] and chronic low back pain [12,25], little is known about the mechanism of chronic pain in SCD. This knowledge gap is in contrast to acute pain secondary to vaso-occlusion, which is well-described [2,3]. A multi-cellular process that involves sickle red cells, white cells, platelets, coagulation factors and the endothelium, vaso-occlusion causes episodic bouts of ischemia and injury that occur, on average, once per year in a child with SCD [2,3]. In the pediatric population, these “crises” are typically self-limited and, between episodes, children are pain-free [26]. However, the pain experience can change as patients with SCD age. In 50% of patients, these intermittent episodes of pain evolve into a chronic pain syndrome [1]. It is this chronic pain syndrome that underlies many acute care admissions in adults and is so confusing to clinicians [26–28]. What is frequently observed is not pain due to avascular necrosis, or another anatomical lesion, but widespread pain, often with accompanying hyperalgesia and allodynia. This clinical picture has led investigators to speculate that sensitization, or enhanced nociception, plays a role in SCD chronic pain [28–32].

In other pain conditions, such as fibromyalgia [33–36], diabetic neuropathy [37,38], and chronic low back pain [20,23,25,39], patients with sensitization not only display hyperalgesia or allodynia but also demonstrate synaptic changes on rsfMRI of the brain. In fact, there is a growing literature in these pain disorders that demonstrate abnormal connectivity between pain centers of the brain and areas involved in memory, emotion, and introspection [33–39]. Of note, in chronic pain disorders, the directionality of the connectivity (positive or negative) is likely harder to interpret than simply pro- or anti-nociceptive, precisely because of the chronic nature of the process. Although a limitation, these abnormal connections are still thought to be revealing, providing insight into the mechanism of neuro-plasticity, a process by which nerves form new connections in response to environmental stimuli, such as learning a new task [40,41]. In the case of central sensitization, however, the changes are maladaptive (for example, linking an emotive region to a pain region), and in response to pathology: injury, inflammation, and/or persistent pain input [13]. Because these are all features inherent to SCD pathogenesis [3], there is ample rationale that sensitization could cause reorganization of the neural networks in this patient population too. Beyond the fact that many adults display hyperalgesia and allodynia, additional evidence that sensitization occurs in patients with SCD comes from quantitative sensory testing [31,32] as well as limited rsfMRI studies [8,9,14] that show changes in brain connectivity. Although speculative, if present, these maladaptive changes could promote and sustain the chronic pain syndrome seen in patients with SCD. In this case, vaso-occlusion that was previously subclinical might later be perceived as pain, which could explain why the frequency and severity of pain worsens with age, even though vaso-occlusion does not [1,26].

In our study, rsfMRI was used to determine if there was evidence of maladaptive changes in the brains of adults with SCD and chronic pain. Compared to SCD patients without chronic pain, those with chronic pain demonstrated changes indistinguishable from zero, which suggested a breakdown in connectivity between PAG and 9 regions of the brain, including several that are part of the DMN, a network that has been implicated in the pathogenesis of chronic pain [21]. Consisting of the inferior parietal lobule, the posterior cingulate cortex, the precuneus, the hippocampus, the posterior lateral cortex, as well as areas of the medial frontal gyri, the DMN is responsible for what the brain is doing when not attending to a task [42–46]. Although counter-intuitive, emerging data has demonstrated that, even in the absence of a task, the brain is never fully at rest [42]. On the contrary, processes such as introspection and memory are always occurring in the background and it is these functions that are thought to be critical to the pathogenesis of chronic pain [20,21]. If the regions of the DMN become fragmented as a result of chronic pain as has been seen in other populations, the perception of pain can become uncoupled from the need for noxious stimuli and, instead, linked to these “background” functions [20,21]. Given that the patients with SCD and chronic pain in our cohort demonstrated a similar breakdown of functional connectivity, these changes may explain how an intermittent, vaso-occlusion-associated phenomenon could become one that is chronic and may occur with minimal injury or in the absence of it altogether.

Like the DMN, another area that has been implicated in chronic pain is the insula [47]. The insula is postulated to play a role in the integration of motor, emotional, autonomic, and cognitive aspects of pain signals [48–50]. With this complex integration, it has been identified as playing a role in the experience of body self-awareness [51,52], sense of agency (the capacity of an individual to act independently and to make their own free choices) [53], and sense of body ownership [54]. Previous fMRI studies have demonstrated that the insula is activated during experimental pain tasks, and is involved in the conscious determination of pain severity [48–50,55,56]. Interestingly, recent research in patients with fibromyalgia has indicated that the insula may also be critical for DMN modulation, suggesting a possible dual role for the insula in chronic pain [57].

As with any study, ours had limitations. First, several confounders may have affected our results. Mental health [58], opioid use [59], substance abuse [60], and neurodegenerative conditions [61,62], have all been demonstrated to independently affect functional connectivity. Although our eligibility criteria, which precluded patients with known stroke and substance abuse, should have diminished the effect of these confounders, they may not have removed their influence entirely. While we will not be able to address these limitations within the current study, they will be the focus of future studies by our research group. Second, adults with SCD in our cohort had a significantly lower hemoglobin than the healthy controls. Potentially, this difference raises the question of whether the functional changes noted might be driven by anemia [63]. To address this, we performed a regression analysis in which we adjusted for group differences (cases/controls) to examine the effects of hemoglobin. The only location that was common between this regression analysis examining hemoglobin and our case/control analysis was the left occipital gyrus (S1 Fig). Given that others have found no global differences in BOLD signal between patients with SCD and controls at rest [9], these data suggest that the changes in functional connectivity identified in our study are not anemia-driven. Third, while SCD phenotype differs by genotype, we chose to include all genotypes to increase the generalizability of our findings. The phenotype that we focused on, chronic pain, is present in all SCD genotypes. Lastly, as is the case with most rsfMRI studies, ours had a small sample size. The small sample size increases the risk of both type I and type II errors. While true, our findings largely agree with the findings from fibromyalgia and chronic low back pain, suggesting that the differences in connectivity that we did identify are biologically meaningful.

Taken together, our data demonstrate that adult patients with SCD have synaptic changes in the CNS that may contribute to the pathogenesis of chronic pain. Like those with fibromyalgia and chronic low back pain, functional areas important for memory, emotion, and introspection are altered, suggesting a common mechanism for the development and maintenance of chronic pain across disease states. Regardless of the inciting event, these maladaptive changes remove the sensation of pain from strictly nociceptive pathways, and may explain how pain evolves from acute to chronic.

## Supporting information

S1 Fig Periaqueductal gray (PAG) seed-based functional connectivity (FC) analyses between patients with SCD (SCD) and controls (CN) as a function of hemoglobin. Image shows the results of the two sample t-test pattern based on hemoglobin level. Color is coded based on z-score of the significance. Brain regions with warm color represent the positive connection and cold color represents the autocorrelation with PAG regions. Brain regions are numbered: (1) Left and right medial frontal and superior frontal gyrus, (2) Left middle occipital gyrus, 3) Left and right medial frontal gyrus, Rectal part, (4) Left Thalamus, and (5) Right Thalamus.
(TIF)

## References

[1] Smith WR, Penberthy LT, Bovbjerg VE, McClish DK, Roberts JD, Dahman B, Aisiku IP, Levenson JL, Roseff SD. Daily assessment of pain in adults with sickle cell disease. Ann Intern Med. 2008; 148(2):94–101. 18195334 10.7326/0003-4819-148-2-200801150-00004

[2] Francis RB Jr, Johnson CS. Vascular occlusion in sickle cell disease: current concepts and unanswered questions. Blood. 1991;77(7):1405–1414. 2009364

[3] Platt OS. Sickle cell anemia as an inflammatory disease. J Clin Invest. 2000;106(3):337–338. 10.1172/JCI10726 10930436 PMC314335

[4] Woolf CJ. Central sensitization: implications for the diagnosis and treatment of pain. Pain. 2011; 152(3 Suppl):S2–15. 10.1016/j.pain.2010.09.030 20961685 PMC3268359

[5] Sethna NF, Meier PM, Zurakowski D, Berde CB. Cutaneous sensory abnormalities in children and adolescents with complex regional pain syndromes. Pain. 2007;131:153–16. 10.1016/j.pain.2006.12.028 17329025

[6] Treede RD, Meyer RA, Raja SN, Campbell JN. Peripheral and central mechanisms of cutaneous hyperalgesia. Prog Neurobiol. 1992;38:397–421. 1574584 10.1016/0301-0082(92)90027-c

[7] Cataldo G, Rajput S, Gupta K, Simone DA. Sensitization of nociceptive spinal neurons contributes to pain in a transgenic model of sickle cell disease. Pain 2015;156: 722–730. 10.1097/j.pain.0000000000000104 25630029 PMC4366346

[8] Darbari DS, Hampson JP, Ichesco E, Kadom N, Vezina G, Evangelou I, Clauw DJ, Taylor JG Vi, Harris RE. Frequency of hospitalizations for pain and association with altered brain network connectivity in sickle cell disease. J. Pain 2015;16:1077–1086. 10.1016/j.jpain.2015.07.005 26291276 PMC4986827

[9] Case M, Zhang H, Mundahl J, Datta Y, Nelson S, Gupta K, He B. Characterization of functional brain activity and connectivity using EEG and fMRI in patients with sickle cell disease. Neuroimage Clin. 2016;14:1–17. 10.1016/j.nicl.2016.12.024 28116239 PMC5226854

[10] Cagnie B, Coppieters I, Denecker S, Six J, Danneels L, Meeus M. Central sensitization in fibromyalgia? A systematic review on structural and functional brain MRI. Semin Arthritis Rheum. 2014;44(1):68–75. 10.1016/j.semarthrit.2014.01.001 24508406

[11] Cifre I, Sitges C, Fraiman D, Muñoz MÁ, Balenzuela P, González-Roldán A, Martínez-Jauand M, Birbaumer N, Chialvo DR, Montoya P. Disrupted functional connectivity of the pain network in fibromyalgia. Psychosom Med. 2012;74(1):55–62. 10.1097/PSY.0b013e3182408f04 22210242

[12] Kregel J, Meeus M, Malfliet A, Dolphens M, Danneels L, Nijs J, Cagnie B. Structural and functional brain abnormalities in chronic low back pain: A systematic review. Semin Arthritis Rheum. 2015;45(2):229–37. 10.1016/j.semarthrit.2015.05.002 26092329

[13] Baliki MN, Apkarian AV. Nociception, Pain, Negative Moods, and Behavior Selection. Neuron. 2015;87(3):474–91. 10.1016/j.neuron.2015.06.005 26247858 PMC4529956

[14] Colombatti R, Lucchetta M, Montanaro M, Rampazzo P, Ermani M, Talenti G, Baracchini C, Favero A, Basso G, Manara R, Sainati L. Cognition and the Default Mode Network in Children with Sickle Cell Disease: A Resting State Functional MRI Study. PLoS One. 2016;11(6):e0157090 10.1371/journal.pone.0157090 27281287 PMC4900543

[15] Dampier C, Palermo TM, Darbari DS, Hassell K, Smith W, Zempsky W. AAPT Diagnostic Criteria for Chronic Sickle Cell Disease Pain. J Pain. 2017;18(5):490–498. 10.1016/j.jpain.2016.12.016 28065813

[16] Hemington KS, Coulombe MA. The periaqueductal gray and descending pain modulation: why should we study them and what role do they play in chronic pain? J. Neurophysiol. 2015; 114, 2080–2083. 10.1152/jn.00998.2014 25673745

[17] Linnman C, Moulton E A, Barmettler G, Becerra L, Borsook D. Neuroimaging of the periaqueductal gray: state of the field. Neuroimage. 2012;60, 505–522. 10.1016/j.neuroimage.2011.11.095 22197740 PMC3288184

[18] Coulombe MA, Erpelding N, Kucyi A, Davis KD. Intrinsic functional connectivity of periaqueductal gray subregions in humans. Hum. Brain Mapp.2016;37, 1514–1530. 10.1002/hbm.23117 26821847 PMC6867375

[19] Xie C, Goveas J, Wu Z, Li W, Chen G, Franczak M, Antuono PG, Jones JL, Zhang Z, Li SJ. Neural basis of the association between depressive symptoms and memory deficits in nondemented subjects: resting-state fMRI study. Hum Brain Mapp. 2012;33(6):1352–63. 10.1002/hbm.21291 21618660 PMC3190573

[20] Baliki MN, Geha PY, Apkarian AV, Chialvo DR. Beyond feeling: chronic pain hurts the brain, disrupting the default-mode network dynamics. J. Neurosci. 2008;28,1398–1403. 10.1523/JNEUROSCI.4123-07.2008 18256259 PMC6671589

[21] Baliki MN, Mansour AR, Baria AT, Apkarian AV. Functional reorganization of the default mode network across chronic pain conditions. PLoS One. 2014;9, e10613. 10.1371/journal.pone.0106133 PMC4152156 25180885

[22] Letzen JE, Craggs JG, Perlstein WM, Price DD, Robinson ME. Functional connectivity of the default mode network and its association with pain networks in irritable bowel patients assessed via lidocaine treatment. J. Pain. 2013;14, 1077–1087. 10.1016/j.jpain.2013.04.003 23743257 PMC3791210

[23] Loggia ML, Kim J, Gollub RL, Vangel MG, Kirsch I, Kong J, Wasan AD, Napadow V. Default mode network connectivity encodes clinical pain: an arterial spin labeling study. Pain. 2013;154, 24–33. 10.1016/j.pain.2012.07.029 23111164 PMC3534957

[24] Napadow V, Kim J, Clauw DJ, Harris RE. Decreased intrinsic brain connectivity is associated with reduced clinical pain in fibromyalgia. Arthritis Rheum. 2012;64, 2398–2403. 10.1002/art.34412 22294427 PMC3349799

[25] Tagliazucchi E, Balenzuela P, Fraiman D, Chialvo DR. Brain resting state is disrupted in chronic back pain patients. Neurosci. Lett. 2010;485, 26–31. 10.1016/j.neulet.2010.08.053 20800649 PMC2954131

[26] Ballas SK, Gupta K, Adams-Graves P. Sickle cell pain: a critical reappraisal. Blood. 2012;120(18):3647–56. 10.1182/blood-2012-04-383430 22923496

[27] Ballas SK. Pain management of sickle cell disease. *Hematol Oncol Clin North Am*. 2005;19(5):785–802. 10.1016/j.hoc.2005.07.008 16214644

[28] Tran H, Gupta M, Gupta K. Targeting novel mechanisms of pain in sickle cell disease Blood. 2017;130(22):2377–2385. 10.1182/blood-2017-05-782003 29187376 PMC5709786

[29] Treede RD, Meyer RA, Raja SN, Campbell JN. Peripheral and central mechanisms of cutaneous hyperalgesia. Prog Neurobiol. 1992;38:397–421. 1574584 10.1016/0301-0082(92)90027-c

[30] Brandow AM, Farley R, Panepinto JA. Neuropathic pain in patients with sickle cell disease. Pediatr Blood Cancer. 2014;61:512–517. 10.1002/pbc.24838 24167104 PMC4357477

[31] Ezenwa MO, Molokie RE, Wang ZJ, Yao Y, Suarez ML, Pullum C, Schlaeger JM, Fillingim RB, Wilkie DJ. Safety and utility of quantitative sensory testing among adults with sickle cell disease: Indicators of neuropathic pain? Pain Pract. 2016;16(3):282–93. 10.1111/papr.12279 25581383 PMC4499503

[32] Brandow AM, Stucky CL, Hillery CA, Hoffmann RG, Panepinto JA. Patients with sickle cell disease have increased sensitivity to cold and heat. Am J Hematol. 2013;88:37–43. 10.1002/ajh.23341 23115062 PMC3552380

[33] Ichesco E, Schmidt-Wilcke T, Bhavsar R, Clauw DJ, Peltier SJ, Kim J, Napadow V, Hampson JP, Kairys AE, Williams DA, Harris RE. Altered resting state connectivity of the insular cortex in individuals with fibromyalgia. J Pain. 2014;15(8):815–826.e1. 10.1016/j.jpain.2014.04.007 24815079 PMC4127388

[34] Jarrahi B, Martucci KT, Nilakantan AS, Mackey S. Investigating the BOLD spectral power of the intrinsic connectivity networks in fibromyalgia patients: A resting-state fMRI study. Conf Proc IEEE Eng Med Biol Soc. 2017;2017:497–500. 10.1109/EMBC.2017.8036870 29059918 PMC5966014

[35] Cifre I, Sitges C, Fraiman D, Muñoz MÁ, Balenzuela P, González-Roldán A, Martínez-Jauand M, Birbaumer N, Chialvo DR, Montoya P. Disrupted functional connectivity of the pain network in fibromyalgia. Psychosom Med. 2012;74(1):55–62. 10.1097/PSY.0b013e3182408f04 22210242

[36] Kim JY, Kim SH, Seo J, Kim SH, Han SW, Nam EJ, Kim SK, Lee HJ, Lee SJ, Kim YT, Chang Y. Increased power spectral density in resting-state pain-related brain networks in fibromyalgia. Pain. 2013;154(9):1792–7. 10.1016/j.pain.2013.05.040 23714266

[37] Cauda F, D’Agata F, Sacco K, Duca S, Cocito D, Paolasso I, Isoardo G, Geminiani G. Altered resting state attentional networks in diabetic neuropathic pain. J Neurol Neurosurg Psychiatry. 2010;81(7):806–11. 10.1136/jnnp.2009.188631 19955113

[38] Cauda F, Sacco K, Duca S, Cocito D, D’Agata F, Geminiani GC, Canavero S. Altered resting state in diabetic neuropathic pain. PLoS One. 2009;4(2):e4542 10.1371/journal.pone.0004542 19229326 PMC2638013

[39] Kornelsen J, Sboto-Frankenstein U, McIver T, Gervai P, Wacnik P, Berrington N, et al Default mode network functional connectivity altered in failed back surgery syndrome. JPain2013;14:483–91. 10.1016/j.jpain.2012.12.018 23498869

[40] Apkarian AV, Baliki MN, and Geha PY. Towards a theory of chronic pain. Prog. Neurobiol. 2009;87, 81–97. 10.1016/j.pneurobio.2008.09.018 18952143 PMC2650821

[41] Apkarian AV, Hashmi JA, and Baliki MN. Pain and the brain: specificity and plasticity of the brain in clinical chronic pain. Pain. 2011;152 (3, Suppl), S49–S64. 21146929 10.1016/j.pain.2010.11.010 PMC3045648

[42] Raichle ME, MacLeod AM, Snyder AZ, Powers WJ, Gusnard DA, Shulman GL. A default mode of brain function. Proc. Natl. Acad. Sci. U. S. A. 2001;98, 676–682. 10.1073/pnas.98.2.676 11209064 PMC14647

[43] Shulman GL, Corbetta M, Buckner RL, Fiez JA, Miezin FM, Raichle ME, Petersen SE. Common blood flow changes across visual tasks: I. Increases in subcortical structures and cerebellum but not in nonvisual cortex. J. Cogn. Neurosci. 1997;9, 624–647. 10.1162/jocn.1997.9.5.624 23965121

[44] Cavanna AE, Trimble MR. The precuneus: a review of its functional anatomy and behavioural correlates. Brain. 2006;129, 564–583. 10.1093/brain/awl004 16399806

[45] Spreng RN, Grady CL. Patterns of brain activity supporting autobiographical memory, prospection, and theory of mind, and their relationship to the default mode network. J. Cogn. Neurosci. 2010;22, 1112–1123. 10.1162/jocn.2009.21282 19580387

[46] Buckner RL, Andrews-Hanna JR, Schacter DL. The brain’s default network: anatomy, function, and relevance to disease. Ann. N. Y. Acad. Sci. 2008;1124, 1–38. 10.1196/annals.1440.011 18400922

[47] Napadow V., LaCount L., Park K., As-Sanie S., Clauw D.J., Harris R.E. Intrinsic brain connectivity in fibromyalgia is associated with chronic pain intensity. Arthritis Rheum. 2010;62:2545–2555. 10.1002/art.27497 20506181 PMC2921024

[48] Baliki MN, Geha PY, Apkarian AV. Parsing pain perception between nociceptive representation and magnitude estimation. J. Neurophysiol. 2009;101 (2): 875–87. 10.1152/jn.91100.2008 19073802 PMC3815214

[49] Ogino Y, Nemoto H, Inui K, Saito S, Kakigi R, Goto F. Inner experience of pain: imagination of pain while viewing images showing painful events forms subjective pain representation in human brain. Cereb. Cortex. 2007;17 (5): 1139–46. 10.1093/cercor/bhl023 16855007

[50] Song GH, Venkatraman V, Ho KY, Chee MW, Yeoh KG, Wilder-Smith CH. Cortical effects of anticipation and endogenous modulation of visceral pain assessed by functional brain MRI in irritable bowel syndrome patients and healthy controls. Pain. 2006;126 (1–3): 79–90. 10.1016/j.pain.2006.06.017 16846694

[51] Karnath HO, Baier B, Nägele T. Awareness of the functioning of one’s own limbs mediated by the insular cortex?. J. Neurosci. 2005;25 (31): 7134–8. 10.1523/JNEUROSCI.1590-05.2005 16079395 PMC6725240

[52] Craig AD. How do you feel—now? The anterior insula and human awareness. Nature Reviews Neuroscience. 2009;10 (1): 59–70. 10.1038/nrn2555 19096369

[53] Farrer C, Frith CD. Experiencing oneself vs another person as being the cause of an action: the neural correlates of the experience of agency. NeuroImage. 2002;15 (3): 596–603. 10.1006/nimg.2001.1009 11848702

[54] Tsakiris M, Hesse MD, Boy C, Haggard P, Fink GR. Neural signatures of body ownership: a sensory network for bodily self-consciousness. Cereb. Cortex. 2007;17 (10): 2235–44. 10.1093/cercor/bhl131 17138596

[55] Bär K-J, Berger S, Schwier C, Wutzler U, Beissner F. Insular dysfunction and descending pain inhibition in anorexia nervosa. Acta Psychiatr Scand. 2013;127: 269–278. 10.1111/j.1600-0447.2012.01896.x 22747702

[56] Taylor KS, Seminowicz DA, Davis KD. Two systems of resting state connectivity between the insula and cingulate cortex. Hum Brain Mapp. 2009;30:2731–45. 10.1002/hbm.20705 19072897 PMC6871122

[57] Hsiao FJ, Wang SJ, Lin YY, Fuh JL, Ko YC, Wang PN, Chen WT. Altered insula-default mode network connectivity in fibromyalgia: a resting-state magnetoencephalographic study. J Headache Pain. 2017;18(1):89 10.1186/s10194-017-0799-x 28831711 PMC5567574

[58] Bluhm R, Williamson P, Lanius R, et al Resting state default-mode network connectivity in early depression using a seed region-of-interest analysis: decreased connectivity with caudate nucleus. Psychiatry Clin Neurosci 2009;63(6):754–761. 10.1111/j.1440-1819.2009.02030.x 20021629

[59] Khalili-Mahani N, Chang C, van Osch MJ, et al The impact of “physiological correction” on functional connectivity analysis of pharmacological resting state fMRI. Neuroimage 2013;65:499–510. 10.1016/j.neuroimage.2012.09.044 23022093

[60] Tanabe J, Nyberg E, Martin LF, et al Nicotine effects on default mode network during resting state. Psychopharmacology (Berl) 2011;216(2):287–295. 21331518 10.1007/s00213-011-2221-8 PMC3486925

[61] Tessitore A, Esposito F, Vitale C, et al Default-mode network connectivity in cognitively unimpaired patients with Parkinson disease. Neurology 2012;79(23):2226–2232. 10.1212/WNL.0b013e31827689d6 23100395

[62] Sheline YI, Raichle ME. Resting state functional connectivity in preclinical Alzheimer’s disease. Biol Psychiatry 2013;74(5):340–347. 10.1016/j.biopsych.2012.11.028 23290495 PMC3537262

[63] Coloigner J, Kim Y, Bush A, Choi S, Balderrama MC, Coates TD, ONeil SH, Lepore N, Wood JC. Contrasting resting-state fMRI abnormalities from sickle and non-sickle anemia. Plos One. 2017;12(10):e0184860 10.1371/journal.pone.0184860 28981541 PMC5628803

