# Supplementary figures and images for: Chronic pain in adults with sickle cell disease is associated with alterations in functional connectivity of the brain
Source: PLoS One. 2019 May 20;14(5):e0216994. doi: 10.1371/journal.pone.0216994 (PMC6527293; doi:10.1371/journal.pone.0216994)

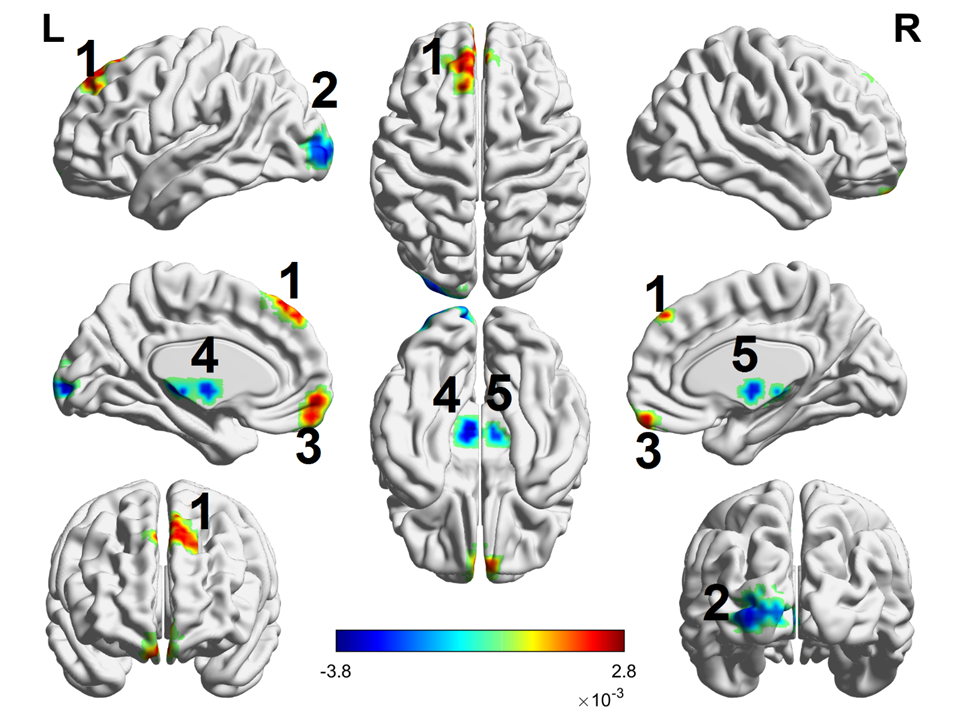

Supplement: S1 Fig — Image shows the results of the two sample t-test pattern based on hemoglobin level. Color is coded based on z-score of the significance. Brain regions with warm color represent the positive connection and cold color represents the autocorrelation with PAG regions. Brain regions are numbered: (1) Left and right medial frontal and superior frontal gyrus, (2) Left middle occipital gyrus, 3) Left and right medial frontal gyrus, Rectal part, (4) Left Thalamus, and (5) Right Thalamus. (TIF) [file pone.0216994.s001.tif]
